# A Multilevel Analysis of Neighbourhood, School, Friend and Individual-Level Variation in Primary School Children’s Physical Activity

**DOI:** 10.3390/ijerph16244889

**Published:** 2019-12-04

**Authors:** Ruth Salway, Lydia Emm-Collison, Simon J. Sebire, Janice L. Thompson, Deborah A. Lawlor, Russell Jago

**Affiliations:** 1 Centre for Exercise, Nutrition & Health Sciences, School for Policy Studies, University of Bristol, 8 Priory Road, Bristol BS8 1TZ, UK; ruth.salway@bristol.ac.uk (R.S.); lydia.emm-collison@bristol.ac.uk (L.E.-C.); simon.sebire@bristol.ac.uk (S.J.S.); 2 School of Sport, Exercise and Rehabilitation Sciences, University of Birmingham, Birmingham B15 2TT, UK; j.thompson.1@bham.ac.uk; 3 MRC Integrative Epidemiology Unit at the University of Bristol, Oakfield House, Oakfield Grove, Bristol BS8 2BN, UK; d.a.lawlor@bristol.ac.uk; 4 Population Health Sciences, Bristol Medical School, University of Bristol, Canynge Hall, Whiteladies Road, Bristol BS8 2PS, UK

**Keywords:** physical activity, children, schools, MVPA

## Abstract

Physical activity is influenced by individual, inter-personal and environmental factors. In this paper, we explore the variability in children’s moderate-to-vigorous physical activity (MVPA) at different individual, parent, friend, school and neighbourhood levels. Valid accelerometer data were collected for 1077 children aged 9, and 1129 at age 11, and the average minutes of MVPA were derived for weekdays and weekends. We used a multiple-membership, multiple-classification model (MMMC) multilevel model to compare the variation in physical activity outcomes at each of the different levels. There were differences in the proportion of variance attributable to the different levels between genders, for weekdays and weekends, at ages 9 and 11. The largest proportion of variability in MVPA was attributable to individual variation, accounting for half of the total residual variability for boys, and two thirds of the variability for girls. MVPA clustered within friendship groups, with friends influencing peer MVPA. Including covariates at the different levels explained only small amounts (3%–13%) of variability. There is a need to enhance our understanding of individual level influences on children’s physical activity.

## 1. Introduction

Physical activity in children is associated with positive health outcomes, including a lower risk of cardiometabolic diseases and improved psychological well-being [1,2]. It is recommended that children over the age of 5 years engage in an average of 60 min of moderate-to-vigorous physical activity (MVPA) per day [3,4,5], yet evidence suggests that many children do not achieve this level of physical activity [6]. Strategies to increase children’s physical activity participation through targeting individual, interpersonal and school-based characteristics have had limited effects [7,8]. Thus, there is a need for a greater understanding of the factors that are associated with children’s physical activity.

Physical activity is a complex behaviour influenced by many individual, inter-personal, environmental and policy level factors [9]. These different levels are consistent with the socio-ecological model, which suggests that each of the different levels impact on behavior, and that the different levels interact [10]. It is noticeable that most studies have focused on the individual level, with factors including sex, age, perceived barriers to activity and motivation consistently associated with physical activity levels in children [11]. Parental factors, including a higher socio-economic position and parental support of physical activity, such as encouragement or the provision of transport, have also been shown to be associated with child MVPA [12]. Children’s physical activity participation may be influenced by the activity levels of their close friends [13,14], and several studies have shown that, during childhood and adolescence, friends engage in similar levels of activity [15,16]. Studies using social network analysis, where the full friendship network is modelled, show weak-to-moderate associations between children’s self-reported [17,18,19,20,21,22] and objectively-assessed MVPA [23,24,25,26], and there is some evidence indicating potential gender differences [22,26]. At the broader school-level, factors such as support for active transportation, physical activity policies, and facilities, have been shown to be associated with physical activity both in and outside of school time [27,28].

Multilevel models have been increasingly used to explicitly model complex hierarchical structures, where children within households are nested within schools and/or neighbourhoods. This type of model is especially common with school-based cluster study designs, where children are nested within schools, as it adjusts standard errors to account for clustering. However, such models also allow description and comparison of the amount of variability at the different levels. A multilevel model combines fixed effects, which are known covariates at different levels that explain physical activity, and random effects that partition the residual variation (that is, the variability due to unknown factors) into variability at the different levels.

Evidence using multi-level modelling consistently shows that children’s physical activity levels vary between schools, with the school accounting for between 2% and 10% of the total variance in daily MVPA [27,28,29,30,31,32,33,34,35], indicating that the school environment can influence children’s physical activity. Most studies have used self-reported physical activity outcomes, but where objective measures of MVPA have been used, the between-school variance is higher, at around 6%–18% [27,31]. Some studies have looked simultaneously at school- and class-level variation to determine the proportion of variability attributable to each level. These studies have shown that variation in physical activity is seen at both the school- and class-level, but that there is greater variation at the class-level for physical activity both within and outside of school [31,35]. These differences remain after adjusting for individual demographics, such as age, gender, ethnicity and parent education level [35], indicating that specific aspects of the school or classroom environment have the potential to influence physical activity participation. While the evidence cited earlier suggests that both peer groups and neighbourhoods vary, we are aware of no studies that have explicitly described between-friend or between-neighbourhood variations. It is also not clear how known factors and unknown variability at multiple levels are related, for example, whether differences between schools are due to school-level factors, demographics, structures such as classes or friendship groups within the school, or a combination of all of these.

The aim of this paper is to explore the variability in children’s accelerometer-measured MVPA at five different levels of the hierarchical levels of socio-ecological model: individual, friendship groups (at two levels), school and neighbourhood, both before and after accounting for child, parent and household factors. We identify the proportion of total variability that can be attributed to each level. These analyses are intended to enable a better understanding of how different factors explain variability at the different levels, and where the residual variability (due to unknown factors) lies. This will enable us to identify the levels within the hierarchical structure that are most important to the physical activity patterns of primary school aged children and help to inform the development of complex interventions targeted at these different levels to increase children’s physical activity at a population level.

## 2. Materials and Methods

B-PROACT1V is a longitudinal study that examined the physical activity and sedentary behaviours of primary school children, aged 5–11 years, and their parents. The study involved three waves of data in Year 1, Year 4 and Year 6 and are described in detail elsewhere [36,37,38]. Participants were recruited from 57 schools in the southwest of England. In this paper we use data collected from Phase 2 in 2015–2016, when the children were aged 8–9 (n = 1223), and Phase 3 in 2017–2018, aged 10–11 (n = 1296). The study received ethical approval from the School of Policy Studies Ethics Committee at the University of Bristol, United Kingdom (UK), and written parental consent was obtained from all participants.

### 2.1. Accelerometer Data

Children and at least one parent of all children in the study wore a waist-worn ActiGraph wGT3X-BT accelerometer (Actigraph LLC, Pensacola, Florida, USA) for three weekdays and two weekend days. Accelerometer data were processed using Kinesoft (v3.3.75; Kinesoft, Saskatchewan, SK, Canada) and analysis was restricted to those children who provided at least two days of valid weekday data and one valid weekend day. This approach ensured that it was possible to conduct separate analyses of weekdays and weekends for the same participant. A valid day was defined as at least 500 min of data, after excluding intervals of ≥60 min of zero counts, allowing up to two minutes of interruptions [6]. Data were recorded at 10 s intervals and characterised as sedentary, light or MVPA using population-specific cut points for children [39] and adults [40]. The average number of MVPA minutes per weekday and weekend day were derived for each child.

### 2.2. Neighbourhood

Each child was allocated to the Lower Layer Super Output Area (LSOA) based on their home postcode. LSOAs are Census-based geographical areas of similar population size, with an average population of 1500, and were used in this study to capture local neighbourhoods. The measure is population-based rather than area-based, but in the current study the average area of an LSOA is 2.3 km^2^ and constitutes a local area that is typically within walking or cycling distance. Variability between neighbourhoods might reflect local differences in facilities or infrastructure. In addition, we included LSOA population density and an LSOA-based measure of deprivation, based on the summary English Indices of Deprivation (IMD; http://data.gov.uk/dataset/index-of-multiple-deprivation), with higher IMD scores indicating a greater level of deprivation.

### 2.3. Friendship Networks

At ages 9 and 11, children were asked to name up to four of their closest friends within their school and year group. These nominations were matched with other participants in the study to form friendship networks, described via an undirected adjacency matrix [41]. This defines a friendship tie between two children in the study if either child nominated the other. To investigate variation at the friend level, we derived clique membership for cliques of minimum size two (clique-2) and of minimum size three (clique-3). These represent ties between pairs of friends (dyads) and between a group of three friends (triads), respectively, who are all linked together via friendship ties. There were insufficient cliques of size four to include as an additional level in the model. Weight matrices were constructed with each clique membership equally weighted; so, for example, if an individual is a member of three cliques, the weight is 1/3 for each clique.

### 2.4. Child Characteristics

Parents reported child gender and date of birth. Child height and weight were recorded to the nearest 0.1 cm and 0.1 kg by trained fieldworkers at each time point, and body mass index (BMI) was calculated and converted to an age- and sex-specific standard deviation score (BMI z-score) based on UK reference curves [42]. Children completed a questionnaire at the time when accelerometers were issued. The questionnaire included questions on how often they attended a sport or exercise club at school, a sport or exercise club outside of school, and how often they played with friends and family outside near their home. These were recoded as Never, 1–2 days and 3+ days. At age 11 only, they were additionally asked how they travelled to and from school on each day. These were coded as either active or non-active travel and summarised as the number of days in the week that the child used active travel for at least one journey.

### 2.5. Parental Characteristics

The parent who wore the accelerometer completed a questionnaire, which included information on their gender, date of birth, self-reported height, weight and the highest education qualification of anyone in the household. Where education data were missing (n = 215 (17%), we used a response from earlier phases of the study if available. This was coded as ‘Up to University Degree/equivalent’ and ‘University Degree/equivalent or higher’. Height and weight were used to derive parent BMI. Parents completed the logistic support (three items, e.g., I take my child places where he/she can be physically active), parental modelling (three items, e.g., I encourage my child to be physically active by leading by example), and use of community resources (three items e.g., I encourage my child to use resources in our neighbourhood to be physically active) subscales of the Activity Support Scale [43]. Items were scored on a four-point Likert type scale (1 = strongly disagree to 4 = strongly agree) and, for analysis, scores were averaged within subscales.

### 2.6. Statistical Analysis

**Figure 1:**
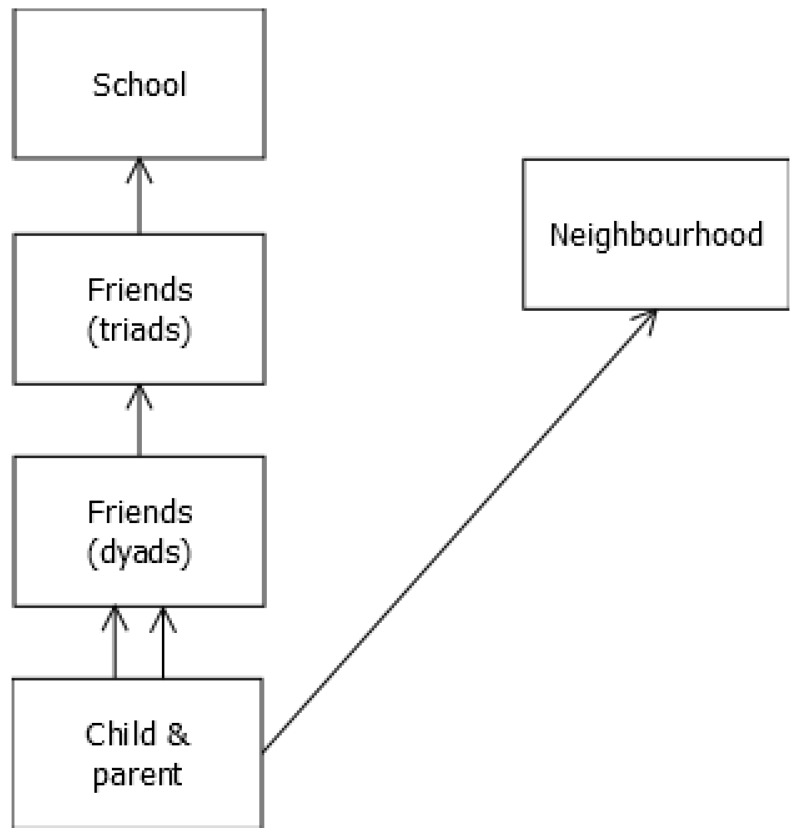
Classification diagram for the multiple-membership multiple-classification model. An arrow indicates a nested relationship; a double arrow indicates multiple membership.

Key variables for child and parent characteristics were summarised for all children, as well as by neighbourhood and school. To compare the variation in physical activity outcomes at different levels, we used a multiple-membership, multiple-classification model (MMMC) for social network dependencies [44,45]. This statistical approach allowed us to include all five sources of variability in a single multilevel model with five levels, summarised in the classification diagram in Figure 1: children (level 1) belong to multiple dyad friendship groups (level 2, multiple-membership) nested within friendship triads (level 3), nested within schools (level 4) and neighbourhoods (level 5, cross-classified). In practice, children are additionally nested within families, but as the study collected data on only one child per family, we were unable to separate the two levels in the current analysis, and so child, parent and family characteristics are all modelled at level 1. The friendship network is defined by two (multiple-membership) network subgroups: membership in clique-2s, which captures the variability between friendship dyads, and clique-3s, which captures variability between friendship triads who are all connected to each other (thus, some of the dyads are nested within triads). Each child may belong to multiple friendship groups. Additionally, children belong to neighbourhoods, but schools and friendship groups are not assumed to be nested within these neighbourhoods (multiple-classification).

We analysed MVPA at ages 9 and 11 years on weekdays and weekend days separately. We were unable to look at the transition between the two time points because there was very little overlap in the friendship networks, and so it was not possible to separate between-friendship group variation and between-timepoint variation. We considered three models that explore different fixed and random effects. Fixed effect terms explain differences in MVPA in terms of known covariates, similar to standard regression models, and may explain variation at any or all levels of the multilevel structure. For example, child gender explains individual variation in MVPA, but it may also explain some of the similarities within friendship groups, as children are more likely to choose friends of the same gender. Random effect terms describe the structure of any remaining variation which is unaccounted for by the fixed terms and can thus identify clustering at different levels. The three models were as follows (full model equations are given in Supplementary Material, File S1).

#### 2.6.1. Model 1: Variance Component Models (No Fixed Terms, Random Intercepts)

This model describes the percentage of total variation in MVPA at the neighbourhood, school, friendship and individual levels. We fitted a variance component model which included an intercept term only in the fixed effects, and random variation at the neighbourhood, school, triad, dyad and individual levels. As children can belong to multiple friendship networks, we calculated the average contribution to the total variance for dyad and triad networks [45] and used this to estimate the share of the total variation at each level.

#### 2.6.2. Model 2: Gender Random Slopes Model (Gender as Fixed Effect, and Gender Random Slope)

Levels and patterns of MVPA differ by gender, and friendships are more likely between children of the same gender. To investigate whether the percentage of variation at each level differed for boys and girls, we included gender both as a fixed effect (to account for gender differences in overall average MVPA) and as a random effect at all levels (to allow the amount of remaining unexplained variation present at each level to differ by gender). This model accounts for any variation at the different levels which is due to clustering by gender, and then estimates the amount of the remaining variation at each level for girls and boys separately.

#### 2.6.3. Model 3: Full Model (Model 2 with Child, Parent, School and Neighbourhood Characteristics as Fixed Effects)

We investigated how much of the variation at different levels was explained by child characteristics (age, BMI z-score, active travel (age 11 only) and participation in school clubs, out-of-school clubs and playing out in the neighbourhood), parental characteristics (age, gender, BMI, household education, weekday/weekend MVPA, and logistical support, parental modelling and use of community resources scales), school size and, additionally, neighbourhood characteristics (IMD, population density), by adding them to Model 2 as fixed effects. The random effects are the same as in Model 2 and describe how much of the residual variation is attributable to each level.

All analyses were performed in Stata V.15.0.29 and MLwiN v3.01 [46] within Stata (StataCorp LL, College Station, TX, USA), via the runmlwin command [47]. Model parameters were estimated using Markov chain Monte Carlo (MCMC), an algorithm that samples a ‘chain’ of correlated parameter values from the joint posterior distribution, and estimates parameters based on summaries of the sample. We report the deviance information criterion (DIC) [48] as a measure of model fit, with a lower DIC indicating a better-fitting model. Further technical details are provided in Supplementary Material (File S1).

#### 2.6.4. Missing Data

At age 9, 1223 children took part, of whom 1077 had valid weekday accelerometer data and 960 had valid weekend data. At age 11, there were 1296 children in total, 1129 with valid weekday and 976 with valid weekend accelerometer data. Only 1% at each time point were missing friendship network data, as this was collected at the time of accelerometer issue. This analysis includes all children who provided data at the relevant time point, including those who listed no friends within the study. Multiple imputation is often used to deal with missing data to maximise the information and potentially increase the statistical power and precision of estimates, but it is computationally difficult with such a complex multilevel model. As a result, we have restricted analysis to only those children who have complete data on all child, parent and neighbourhood covariates: 769 on weekdays and 664 on weekends at age 9, and 735 on weekdays and 585 at weekends at age 11. The majority of missing data was due to non-completion of parent questionnaires.

Missing data in multilevel models can bias estimates of both fixed and random effects unless data are missing completely at random. The treatment of missing data on complex models is a relatively new area of research [49], so it is difficult to anticipate the extent of bias in the current study, where we consider a complex multilevel model with random slopes, and we are interested primarily in estimating the random effects. Moreover, problems with missing data are exacerbated when working with friendship networks, because omitting incomplete cases from analysis disproportionately affects the friendship networks as all ties involving these children would be omitted. This results in very sparse networks and reduces the amount of friendship network variability it is possible to detect [50]. Simulations [51] have suggested that listwise deletion of cases with missing values tends to overestimate variance components, with bias increasing for larger amounts of clustering. However, these simulations found that estimates of random slopes were practically unbiased when sample sizes were sufficiently large, and the authors concluded that listwise deletion was worth considering when the slope variance is of particular importance. In Models 2 and 3, this corresponds to the gender difference random effects, which are of key interest. We therefore used listwise deletion in all our models, but to explore the impact of missing covariate data we reran Models 1 and 2 using the complete data for comparison. Thus, while we recognise the limitation of the chosen approach, it is useful as it provides unique information on the potential of the factors at the different levels to explain some of the variation in children’s physical activity.

## 3. Results

There was considerable variability in MVPA between schools (Table 1), with average MVPA more than doubling between the lowest and highest schools on a weekday (38.6 versus 89.4 min per day) with comparable findings for weekend days. Missing data are described in Table 2. MVPA missing data ranged from 12%–13% for weekdays and 22%–25% for weekends, and parent characteristics and accelerometer missing data ranged from 11%–23%.

Table 3 and Table 4 report the percentage of the total variation in MVPA attributable to known covariates (fixed effects) and random variation at the different levels for age 9 and age 11, respectively. For all models, most of the variation in MVPA was at the individual level, that is, it was not explained by covariates and did not exhibit clustering at any level of the hierarchical structure. Model 1 explored the amount of total variation at each level, and showed patterns differing between weekdays and weekends and between age 9 and age 11. Between-school variation accounted for around 12%–13% of the total MVPA on weekdays, with less clustering on weekends at age 9 (6%). There was very little between-friendship group variation at age 9, but more clustering within friendship groups at age 11, especially on weekdays. On weekdays, the majority of this between-friend variation was clustering within dyads, but on weekends it was dominated by triads. There was very little between-neighbourhood variation. At age 11, more of the total variation was attributable to clustering within the hierarchical model structure, especially at the friendship level.

In Model 2, including gender as a fixed effect explained some of the variance at all levels, especially clustering within friendships, but there were strong differences in the patterns of remaining variation by gender. The DIC indicated a large improvement in the model fit when allowing variation to differ by gender. However, as with Model 1, the largest proportion of residual variability in MVPA remained at the individual level, accounting for around a third to half of the total for boys (36%–51%) and over two thirds for girls (60%–78%), with the remainder clustering at other levels of the hierarchical structure. At age 9, both genders showed similar clustering at the school level within the week (14%–16%), but between-school variation at weekends was over twice as much for boys than for girls (19% vs. 6%). A larger proportion of the variability was attributed to friendships for boys than girls, especially at weekends, accounting for 23% of the total on weekdays (combining dyads and triads) and 30% on weekends, compared to 12% and 11%, respectively, for girls. At age 11, both genders showed similar clustering at school level, with a higher proportion of between-school variability on weekdays compared to weekends (16% vs. 10% for boys and 16% vs. 9% for girls).

A larger proportion of the weekday variability was at the friendship group level for both boys and girls (40% and 20% respectively), compared to age 9. On weekdays, clustering was stronger between friendship dyads, but on weekends slightly more variability was attributed to friendship triads. At age 11, neighbourhood accounted for 16% of the total variation in boys’ weekend average MVPA, but for all other age, gender and day combinations, neighbourhood-level variation accounted for relatively small amounts of the total (3%–6%).

To explore the impact of missing covariate data, we reran Models 1 and 2 on the complete data (Tables S1 and S2, Supplementary Material). This suggested that, at age 9, the percentage of variation at the school and neighbourhood levels was slightly overestimated in the presence of the missing data, while, at age 11, the percentage of variation at the dyad friendship level was slightly overestimated. Thus, the residual individual variation reported in Table 3 and Table 4 might, therefore, be higher than estimated.

**Figure 2:**
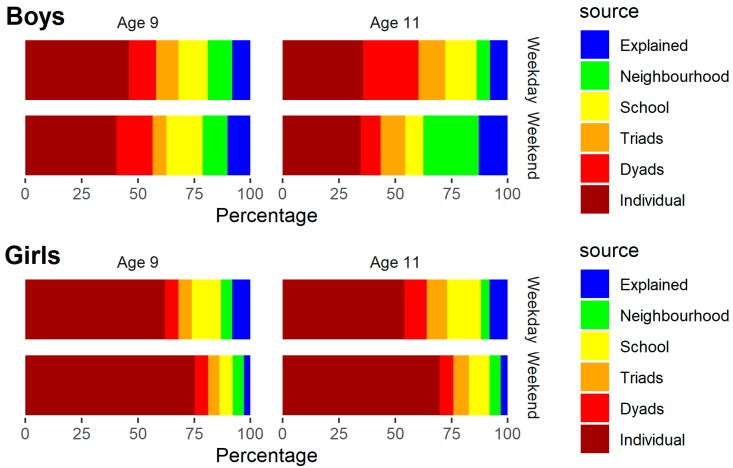
Percentage of total variation from different sources by gender (Model 3).

Model 3 included child, parent and neighbourhood covariates. The DIC indicated a slight improvement in model fit, and both child and parental characteristics were associated with MVPA. However, the covariates explained only 3%–13% of the total variation, and this was mostly individual-level variation. Figure 2 shows the percentage of total variation explained by the covariates, and residual variation at each level for girls and boys, based on Model 3. Estimates for the fixed effects are given in Tables S3 and S4 (Supplementary Material).

## 4. Discussion

A summary of the main findings from this paper is presented in Table 5, which highlights the important additions to the knowledge base of children’s physical activity and how they relate to the existing evidence base. Central among these findings is the new evidence that the largest source of variation in children’s physical activity operates at the individual level, accounting for two thirds of the variability for girls and half of the total residual variability for boys. Thus, the key finding of our study is that there are still important individual-level characteristics associated with children’s physical activity that we have not identified in this study, despite including a large number of variables at different levels. There have been a large number of interventions designed to increase children’s physical activity, yet the majority show, at best, only small increases, equivalent to an increase of around 4 min of MVPA per day [52,53,54]. Our findings suggest that one reason for this could be that we simply do not understand enough about the factors that influence physical activity. This indicates a need for further research to better identify and understand such factors before we are able to develop more effective interventions [55]. This finding suggests that, in addition to the current zeitgeist of focusing on structural and system level influences [9], there is also an urgent need to enhance our understanding of individual level influences on children’s physical activity and that strategies to understand and then change all levels are needed to help more children to be physically active.

The data presented in this paper show that the between school variability was around 14%–16% of the total residual variation on weekdays. The between-friend variability increases between ages 9 and 11 from around 23%–30% to 29%–40% for boys and 11%–12% to 16%–20% for girls. These findings highlight the critical role that friends play on physical activity and how the importance of friends and friendship groups increases as children age. Collectively, these findings suggest that friend- and peer-focused strategies for behaviour change have considerable potential. Such work is ongoing, and a 2012 review highlighted 23 peer and friend-based physical activity studies. However, the majority of these were descriptive, cross-sectional designs that used self-reported measures of physical activity [56]. As such, this is an area that would benefit from greater attention, using more robust measures of physical activity, and could provide a fruitful means for developing new behaviour change interventions.

There were substantial differences between girls and boys in terms of the amount of total variability, the level at which it clustered and the amount explained by known factors. The analyses also showed that the important predictors of physical activity differed with age and for weekday versus weekend days. There was more variability in MVPA among boys than girls, but more of this variability was explained by known factors (i.e., in this study, BMI, active travel, participation in sports clubs, parental physical activity, age, BMI and parental support), and a larger proportion of the residual variation clustered within the hierarchical structure. These results therefore suggest that the factors studied in this study explain more about the physical activity of boys than girls. Studies consistently show that boys are more active than girls at all ages [6,36,38,57], but there is little evidence that the effect of physical activity behaviour change interventions differs for boys and girls [52], with generally limited impacts for both genders. It may therefore be the case that a greater understanding of the factors that influence children’s physical activity is required to promote effective behaviour change programs and, as such, the data from this paper highlight a particular need to look more closely at the individual level predictors of girls’ physical activity.

Weekends were different to weekdays, with nearly twice as much total variability at weekends, a smaller proportion explained by known factors, and less clustering of the residual variation. We have previously found [57] that those children who are most inactive tend to be even more so at weekends, highlighting the importance of weekend physical activity. Current research in this area has been dominated by school-based interventions [8,52] with little attention on weekend or other non-school periods, such as school holidays. This is likely due to the relative ease of recruitment in schools and difficulties recruiting representative samples outside of schools. This finding highlights a need to develop strategies to understand the factors that impact physical activity at weekends and during school holidays, and to use this information to develop behaviour change programs for these settings.

### Methodological Strengths and Limitations

The analysis conducted in this paper used a multi-level model to examine the impact of different types of influences on children’s physical activity. This approach facilitated an assessment of what we know and, perhaps more importantly, what we do not know about children’s physical activity. The information on what we do not know is particularly useful, as it can help to guide future exploratory research. We did not, however, assess psychosocial constructs, such as motivation and competence, that have been shown to be important in other studies of children’s physical activity [58,59]. It is important, however, to recognise that there were a number of technical challenges with this type of analysis, and these limit the potential to fully explore the impact of different sources of variation in physical activity. Key among these challenges is missing data. We are not aware of any routine methods to implement multiple imputation of covariates for a complex multilevel model that includes both cross-classified and multiple-membership levels. As a result, we have had to restrict our analysis to complete cases only. This is an important limitation for social network analyses, as omitting one observation potentially omits multiple friendship ties, which results in an overly sparse friendship network. There is currently no consensus about how to deal with missing data in such networks, or how much bias this may introduce in estimates of either fixed or random effects. Sensitivity analyses in the current study suggested that variation at the neighbourhood, school and friendship levels was slightly overestimated in the presence of the missing data. We were also unable to look at longitudinal change between ages 9 and 11. While, in theory, the MMMC model could be extended to a longitudinal model, in practice there was too little overlap between friendship networks at the two timepoints to facilitate these analyses. While some of this is due to changing friendships, a large portion is once again due to missing data. Finally, this model relies on Bayesian MCMC methods, with associated issues of convergence and computational resources. Future work could take advantage of approximation methods, such as integrated nested Laplace approximations [60].

## 5. Conclusions

We found considerable variation in MVPA, with greater variability for boys than for girls and for weekends than for weekdays. The proportion of the variance was attributable to individual level factors, accounting for half of the total residual variability for boys, and two thirds of the variability for girls, rather than due to clustering within neighbourhoods, schools or friendship groups. Including covariates at the different levels explained only small amounts (3%–13%) of the total variability. There is an urgent need to enhance our understanding of individual level influences on children’s physical activity, so that we are able to develop better interventions.

## Figures and Tables

**Table 1:** Individual, friend, school and neighbourhood characteristics.

	Age 9	Age 11
**Individual Level:**		
	% or mean (sd)	% or mean (sd)
% female	55%	52%
BMI z-score	0.35 (1.07)	0.35 (1.16)
Weekday MVPA (min)	62.3 (22.4)	60.6 (23.1)
Weekend MVPA (min)	61.3 (32.0)	53.4 (31.3)
% with a degree or higher	52%	53%
IMD score	15.9 (14.1)	15.4 (14.4)
Parent age	41.3 (6.3)	42.9 (6.0)
Parent BMI	25.9 (4.9)	25.9 (4.8)
Parent weekday MVPA (min)	54.2 (28.9)	54.7 (28.5)
Parent weekend MVPA (min)	42.9 (26.7)	46.7 (29.9)
	mean (min–max)	mean (min–max)
**Friend level:**		
No. of friend ties per child	5 (1–13)	6 (1–14)
No. of dyads ^1^ per child	4 (1–10)	4 (1–10)
No. of triads ^2^ per child	3 (0–13)	3 (0–16)
**School level:**		
Total	47	50
No. participants	26 (7–65)	26 (10–58)
School size	310 (105–1410)	307 (105–1410)
% female	54% (18–76%)	53% (23–73%)
Weekday MVPA (min)	61.8 (38.6–89.4)	59.4 (28.6–83.5)
Weekend MVPA (min)	61.9 (39.8–86.5)	54.1 (32–107.2)
**Neighbourhood level:**		
Total	367	346
No. participants	3 (1–23)	4 (1–115)
Area (km^2^)	2.4 (0.1–48.5)	2.0 (0.1–48.5)
Population density (1000/km^2^)	4.1 (0.04–18.7)	4.4 (0.04–18.8)
% female	56% (0–100%)	54% (0–100%)
Weekday MVPA (min)	60.6 (9.6–164.1)	58.1 (12.4–118.3)
Weekend MVPA (min)	59.0 (7.7–200.2)	52.1 (6.2–177.8)

^1^ dyads: pairs of friends. ^2^ triads: groups of three friends who are all connected with each other. MVPA: Moderate to Vigorous Intensity Physical Activity.

**Table 2:** Number of participants with valid data and the proportion of missing data for key variables.

	Age 9		Age 11	
	N	% Missing	N	% Missing
Total	1223		1296	
LSOA	1208	1%	1181	9%
Friendship network	1210	1%	1289	0.5%
% female	1223	0%	1296	0%
BMI z-score	1217	0.5%	1285	1%
Weekday MVPA (min)	1077	12%	1129	13%
Weekend MVPA (min)	960	22%	976	25%
% with a degree or higher	1125	8%	1191	8%
IMD score	1204	2%	1251	3%
Parent age	975	20%	1064	18%
Parent BMI	951	22%	994	23%
Parent weekday MVPA (min)	1090	11%	1143	12%
Parent weekend MVPA (min)	952	22%	992	23%
Total for weekday analysis	769	37%	735	43%
Total for weekend analysis	664	46%	585	55%

**Table 3:** Percentage of variation in MVPA at age 9.

	Model 1	Model 2		Model 3	
	All	Boys	Girls	Boys	Girls
**Weekday**					
**Total variation**	505.6	569.8	423.7	569.8	423.7
**Explained variation**					
Child, parent, school and neighbourhood factors	-	-	-	8%	8%
**Residual variation**					
Neighbourhood	1%	10%	5%	11%	5%
School	13%	16%	14%	13%	13%
Triads ^1^	2%	10%	6%	10%	6%
Dyads ^1^	1%	13%	6%	12%	6%
Individual	82%	51%	69%	46%	62%
DIC ^2^	6881.4		6758.3		6700.3
**Weekend**					
**Total variation**	1008.7	1386.2	734.2	1386.2	734.2
**Explained variation**					
Child, parent, school and neighbourhood factors	-	-	-	10%	3%
**Residual variation**					
Neighbourhood	6%	10%	6%	11%	5%
School	5%	19%	6%	16%	6%
Triads ^1^	1%	6%	5%	6%	5%
Dyads ^1^	2%	24%	6%	16%	6%
Individual	86%	41%	78%	40%	76%
DIC ^2^	6442.5		6306.9		6287.9

^1^ For friendship levels, we report the average contribution to the total variance. ^2^ lower DIC indicates better model fit.

**Table 4:** Percentage of variation in MVPA at age 11.

	Model 1	Model 2		Model 3	
	All	Boys	Girls	Boys	Girls
**Weekday**					
**Total variation**	510.0	578.9	379.5	578.9	379.5
**Explained variation**					
Child, parent, school and neighbourhood factors	-	-	-	8%	8%
**Residual variation**					
Neighbourhood	1%	6%	4%	6%	4%
School	13%	16%	16%	14%	15%
Triads ^1^	4%	13%	9%	12%	9%
Dyads ^1^	28%	27%	11%	25%	10%
Individual	55%	39%	60%	36%	54%
DIC ^2^	6464.8		6347.8		6294.1
**Weekend**					
**Total variation**	1029.3	1355.7	683.0	1355.7	683.0
**Explained variation**					
Child, parent, school and neighbourhood factors	-	-	-	13%	3%
**Residual variation**					
Neighbourhood	2%	25%	4%	25%	5%
School	12%	10%	9%	8%	9%
Triads ^1^	7%	16%	9%	11%	7%
Dyads ^1^	1%	13%	7%	9%	6%
Individual	79%	36%	71%	35%	69%
DIC^2^	5654.5		5492.7		5478.5

^1^ For friendship levels, we report the average contribution to the total variance. ^2^ lower DIC indicates better model fit.

**Table 5:** Summary of evidence from the literature, the contribution of this study and important unknown information at individual, friendship, school and neighbourhood levels.

Level	Evidence from Literature: Factors Associated with Physical Activity	Contribution of This Study	Important Unknown Information
**Individual** **(and parent)**	Child characteristics: age, gender, BMI, active travel, club attendance, motivation	Child characteristics: gender, BMI z-score, active travel (weekdays), out of school sports clubs	Large amounts of residual variation at the individual level: 35%–46% for boys and 54%–76% for girls.
	Parent characteristics: support, modelling behaviour.	Parent characteristics: age, BMI, MVPA.	This is residual variation that does not cluster within the other levels.
		logistical support (weekday age 9), use of community resources (weekday age 11).	
		The included covariates explained 4%–12% of the total variability, and most of this was at the individual level.	
**Friendship groups**	Friends tend to have similar levels of physical activity	The included covariates accounted for a small amount of the friendship variation on weekends, especially for boys.	Between-friendship variation was around 11%–19% for girls and 20%–37% for boys.
			At age 9, this was split roughly equally between dyad and triad friendships, apart for boys at weekends, where MVPA clustered more within dyads.
			At age 11, weekday MVPA was more likely to cluster in dyads for both boys and girls.
			Boys’ weekend MVPA was dominated by clustering within triads.
**School**	School policies, facilities, support for active travel.	None of the included covariates explained between-school variation.	Between-school variation was around 10%–15% of the total variation.
			Boys and girls similar in the week, but boys showed more clustering at weekends.
**Neighbourhood**	Walkability, traffic, local facilities	None of the included covariates explained between-neighbourhood variation.	Between-neighbourhood variation was small at around 5–10% of the total variation.
			More clustering within neighbourhoods for boys than for girls, especially on weekends at age 11 (25%).

## Supplementary Materials

The following are available online at https://www.mdpi.com/1660-4601/16/24/4889/s1, Table S1: Percentage of variation in MVPA at age 9: Full data, Table S2: Percentage of variation in MVPA at age 11: Full data, Table S3: Fixed effect estimates from Model 3: Age 9, Table S4: Fixed effect estimates from Model 3: Age 11. File S1: Model Specification, MCMC Technical Details, References.
