# Supplementary material for: A Multilevel Analysis of Neighbourhood, School, Friend and Individual-Level Variation in Primary School Children’s Physical Activity
Source: Int J Environ Res Public Health. 2019 Dec 4;16(24):4889. doi: 10.3390/ijerph16244889 (PMC6950546; doi:10.3390/ijerph16244889)
Supplement: Supplementary file 1 [file ijerph-16-04889-s001.pdf]

Supplementary Material

Table S1: Percentage of variation in MVPA at age 9: full data

Table S2: Percentage of variation in MVPA at age 11: full data

Table S3: Fixed effect estimates from Model 3: age 9

Table S4: Fixed effect estimates from Model 3: age 11

File S1:

Model Specification

MCMC Technical Details

References

Table S1: Percentage of variation in MVPA at age 9: Full data

	Model 1	Model 2	
	All	Boys	Girls
Weekday			
**Total variation**	510.5	561.2	433.3
**Residual variation**			
Neighbourhood	0%	7%	3%
School	12%	14%	11%
Triads[1]	1%	8%	5%
Dyads[1]	4%	13%	6%
Individual	83%	58%	75%
DIC[2]	9654.7		9529.1
Weekend			
**Total variation**	1032.2	1340.0	790.8
**Residual variation**			
Neighbourhood	2%	8%	2%
School	3%	14%	3%
Triads[1]	1%	5%	1%
Dyads[1]	0%	24%	0%
Individual	94%	50%	94%
DIC[2]	9368.9		9205.8

[1] For friendship levels, we report the average contribution to the total variance

[2] lower DIC indicates better model fit

Table S2: Percentage of variation in MVPA at age 11: Full data

	Model 1	Model 2	
	All	Boys	Girls
Weekday			
**Total variation**	546.9	614.0	421.6
**Residual variation**			
Neighbourhood	0%	7%	3%
School	14%	18%	16%
Triads[1]	12%	13%	8%
Dyads[1]	14%	17%	7%
Individual	60%	45%	66%
DIC[2]	10036.5		9888.1
Weekend			
**Total variation**	994.1	1232.0	776.7
**Residual variation**			
Neighbourhood	1%	20%	3%
School	10%	10%	9%
Triads[1]	14%	17%	10%
Dyads[1]	1%	7%	4%
Individual	74%	47%	74%
DIC[2]	9374.3		9244.4

[1] For friendship levels, we report the average contribution to the total variance
[2] lower DIC indicates better model fit

Table S3: Fixed effect estimates from Model 3: Age 9

		Weekday		Weekend	
		Coef	95% CI	Coef	95% CI
Intercept		66.4	(25.9, 106.9)	71.5	(10.7, 132.2)
**Child characteristics**					
Female		-14.2	(-17.7, -10.7)	-14.6	(-21.4, -7.9)
Age		1.1	(-2.7, 5.0)	-1.1	(-6.7, 4.6)
BMI z-score		-2.3	(-3.7, -0.9)	-1.2	(-3.4, 1.1)
school sport club	0	0		0	
(days/week)	1-2	2.0	(-1.5, 5.5)	0.4	(-5.1, 5.9)
	>2	5.4	(1.4, 9.5)	-0.8	(-7.3, 5.6)
Non -school sport	0	0		0	
club (days/week)	1-2	-3.7	(-7.6, 0.2)	2.5	(-3.7, 8.7)
	>2	0.4	(-4.0, 4.8)	7.2	(0.4, 14.1)
Playing out	0	0		0	
(days/week)	1-2	-0.5	(-6.3, 5.2)	6.6	(-2.3, 15.6)
	>2	4.4	(-1.2, 9.9)	12.4	(3.7, 21.1)
**Parent characteristics**					
University Degree or higher		-1.5	(-4.5, 1.5)	-0.9	(-5.5, 3.8)
Female		-2.0	(-5.2, 1.3)	0.9	(-4.2, 6.0)
Age		-0.3	(-0.5, -0.05)	-0.6	(-1.0, -0.2)
BMI		0.01	(-0.3, 0.3)	0.00	(-0.5, 0.5)
MVPA (mins)		0.1	(0.1, 0.2)	0.2	(0.1, 0.3)
Logistical support		3.6	(0.7, 6.5)	2.2	(-2.4, 6.8)
Parental modelling		-1.8	(-4.3, 0.7)	1.9	(2.0, 5.9)
Use of community resources		-0.5	(-3.2, 2.2)	0.2	(-4.1, 4.4)
**School characteristics**					
School size (per 100 pupils)		-1.4	(-3.0, 0.1)	-1.3	(-3.3, 0.8)
**Neighbourhood characteristics**					
IMD score		0.00	(-0.1, 0.2)	-0.01	(-0.2, 0.2)
Population density (1000/km^3)		0.2	(-0.4, 0.8)	0.13	(-0.8, 1.0)

Table S4: Fixed effect estimates from Model 3: Age 11

		Weekday		Weekend	
		Coef	95% CI	Coef	95% CI
Intercept		60.1	(9.5, 111.0)	64.3	(-14.1, 143.3)
**Child characteristics**					
Female		-14.3	(-17.9, -10.8)	-14.5	(-20.2, -8.9)
Age		-1.1	(-5.3, 3.0)	-3.3	(-9.7, 3.1)
BMI z-score		-2.8	(-4.1, -1.6)	-1.8	(-4.0, 0.3)
No. days with active travel		0.9	(0.2, 1.6)	0.6	(-0.6, 1.7)
school sport club	0	0		0	
(days/week)	1-2	0.4	(-2.9, 3.7)	3.1	(-2.4, 8.6)
	>2	2.6	(-1.4, 6.6)	8.0	(1.6, 14.5)
Non -school sport	0	0		0	
club (days/week)	1-2	4.7	(0.8, 8.7)	3.1	(-3.3, 9.6)
	>2	7.5	(3.0,11.9)	6.9	(-0.5, 14.3)
Playing out	0	0		0	
(days/week)	1-2	-1.6	(-7.2, 4.0)	-5.9	(-14.8, 3.0)
	>2	-0.1	(-5.6, 5.4)	-2.6	(-11.4., 6.2)
**Parent characteristics**					
University Degree or higher		-0.7	(-3.6, 2.3)	-4.0	(-8.9, 0.9)
Female		4.9	(1.7, 8.0)	4.0	(-1.2, 9.1)
Age		0.3	(-0.01, 0.5)	0.3	(-0.1, 0.8)
BMI		-0.1	(-0.5, 0.2)	-0.3	(-0.9, 0.2)
MVPA (mins)		0.05	(0.00, 0.1)	0.2	(0.1, 0.3)
Logistical support		0.8	(-2.1, 3.7)	3.9	(-0.9, 8.7)
Parental modelling		0.6	(-1.8, 3.0)	0.4	(-3.5, 4.2)
Use of community resources		-3.1	(-5.8, -0.5)	-0.7	(-5.1, 3.7)
**School characteristics**			(-1.4, 1.6)		
School size (per 100 pupils)		0.1		-1.5	(-3.6, 0.6)
**Neighbourhood characteristics**					
IMD score		-0.1	(-0.2, 0.1)	-0.1	(-0.3, 0.1)
Population density (1000/km^3)		-0.1	(-0.7, 0.5)	0.7	(-0.3,1.6)

Supplementary Files:

File S1: Model Details

Model Specification

The model is a multiple-membership multiple-classification model (MMMC) for social network dependencies [1, 2] with children (level 1) belong to multiple clique-2 friendship groups (level 2, multiple-membership) nested within clique-3 (level 3), nested within schools (level 4) and neighbourhoods (level 5, cross-classified). We fit three models, whose specification is given in detail below. We use classification notation [1], which provides a simpler notation than multiple subscript notation and remains readable for more complex non-hierarchical multilevel models.

General Model

Let y_i be the MVPA for individual $i = 1, \ldots, n$. The multilevel model consists of fixed and random terms as follows:

$$y_i = \text{fixed}_i + \text{random}_i$$

Model 1: Variance components

This model describes the percentage of total variation in MVPA at the neighbourhood, school and friendship levels. The fixed effect consists of β_0, an intercept term, and random effects are at the clique-2, clique-3, school and neighbourhood levels. The clique-3 and clique-2 levels are multiple-membership, with clieu-2 nested within clique-3, and schools and cliques are cross-classified with neighbourhood.

$$\text{fixed}_i = \beta_0$$

$$\text{random}_i = u^{(5)}_{\text{nhood}(i)} + u^{(4)}_{\text{school}(i)} + \sum_{j \in \text{clique-3}(i)} w^{(3)}_{i,j}\, u^{(3)}_j + \sum_{j \in \text{clique-2}(i)} w^{(2)}_{i,j}\, u^{(2)}_j + \epsilon_i$$

where clique-2$(i) \subset (1, \ldots, J_2)$, clique-3$(i) \subset (1, \ldots, J_3)$ and

$$\epsilon_i \sim N(0, \sigma^2_\epsilon) \qquad u^{(2)}_j \sim N\left(0, \sigma^2_{u^{(2)}}\right) \qquad u^{(3)}_j \sim N\left(0, \sigma^2_{u^{(3)}}\right)$$

$$u^{(4)}_j \sim N\left(0, \sigma^2_{u^{(4)}}\right) \qquad u^{(5)}_j \sim N\left(0, \sigma^2_{u^{(5)}}\right)$$

Here, clique-$3(i)$ is the set of triads of which i is a member, J_3 is the total number of triads, the term $\sum_{j \in \text{clique-3}(i)} w_{i,j}^{(3)} u_j^{(3)}$ is a weighted sum of clique-3 effects with weights $w_{i,j}^{(3)}$ for individual i in the jth clique, and random effects $u_j^{(3)}$, and the weights sum to 1 for each individual. The set clique-$2(i)$ and terms $\sum_{j \in \text{clique-2}(i)} w_{i,j}^{(2)} u_j^{(2)}$ are defined similarly for clique-2. Finally, school(i) and nhood(i) are the school and neighbourhood respectively to which child i belongs.

Model 2 – Gender random slopes model

This model describes the percentage of total variation in MVPA at the neighbourhood, school and friendship levels separately for boys and girls by adding gender as a fixed effect and as a random coefficient at the neighbourhood, school and clique levels.

$$\text{fixed}_i = \beta_0 + \beta_1 \, \text{girl}_i$$

$$\text{random}_i = u_{0,\text{nhood}(i)}^{(5)} + u_{1,\text{nhood}(i)}^{(5)} \text{girl}_i + u_{0,\text{school}(i)}^{(4)} + u_{1,\text{school}(i)}^{(4)} \text{girl}_i + \sum_{j \in \text{clique-3}(i)} w_{i,j}^{(3)} \left(u_{0j}^{(3)} + u_{1j}^{(3)} \text{girl}_i\right) + \sum_{j \in \text{clique-2}(i)} w_{i,j}^{(2)} \left(u_{0j}^{(2)} + u_{1j}^{(2)} \text{girl}_i\right) + \epsilon_i$$

with

$$\begin{pmatrix} u_{0j}^{(\text{k})} \\ u_{1j}^{(\text{k})} \end{pmatrix} \sim N\left(\begin{pmatrix} 0 \\ 0 \end{pmatrix}, \begin{pmatrix} \sigma_{u0(k)}^2 & \sigma_{u01(k)} \\ \sigma_{u01(k)} & \sigma_{u1(k)}^2 \end{pmatrix} \right)$$

$$\epsilon_i \sim N(0, \sigma_\epsilon^2)$$

for $k = 2, \ldots, 5$, corresponding to the clique-2, clique-3, school and neighbourhood levels.

Model 3 – full model

The full model includes child, parent, school and neighbourhood characteristics as fixed effects:

$$\text{fixed}_i = \beta_0 + \beta_1 \, \text{girl}_i + \sum_{l=1}^{L} \gamma_l x_{il}$$

where $x_{il}, l = 1, \ldots, L$ are the L variables representing the child, parent, school and neighbourhood characteristics, and γ_l are the corresponding coefficients.

The random term is the same as for model 2 above.

MCMC Technical Details

Prior Distributions

We used the MLwiN default non-informative prior distributions for all parameters which express a lack of prior knowledge about the parameters before data collection. These are improper uniform priors (p(β) ∝ 1) for the fixed effects, and weakly informative inverse-Wishart distributions for the variance parameters. Further details can be found in Browne 2016, Chapter 1 [3].

MCMC Estimation

While the chain of sampled parameter values will eventually converge to the required distribution, it may take an initial period (the 'burn-in') to converge, and these values are discarded. Additionally, the sampled values are correlated and if this correlation is high, the chain is said to be slow-mixing and more iterations are required to sample adequately from the full distribution. We assessed convergence of the algorithm via trace plots and by exploring different starting values, and used a burn-in of 20,000 samples for all models. Our models exhibited slow-mixing due to small variance parameters at some levels, and so we based estimation on 1,000,000 iterations to ensure adequate mixing and used hierarchical centring at the highest level, a reparameterisation that can improve mixing of MCMC algorithms [3, 4].

References

1. Browne WJ, Goldstein H, Rasbash J: **Multiple membership multiple classification (MMMC) models**. *Statistical Modelling* 2001, **1**:103-124.
2. Tranmer M, Steel D, Browne WJ: **Multiple-membership multiple-classification models for social network and group dependences**. *J R Statist Soc A* 2014, **177**:439-455.
3. Browne WJ: **MCMC estimation in MLwiN v2.36**: Centre for Multilevel Modelling, Univesity of Bristol; 2016.
4. Gelfand AE, Sahu SK, Carlin BP: **Efficient parametrisations for normal linear mixed models**. *Biometrika* 1995, **82**(3):479-488.
